# Phloem-limited reoviruses universally induce sieve element hyperplasia and more flexible gateways, providing more channels for their movement in plants

**DOI:** 10.1038/s41598-017-15686-x

**Published:** 2017-11-28

**Authors:** Ming-Fang Lv, Li Xie, Xi-Jiao Song, Jian Hong, Qian-Zhuo Mao, Tai-Yun Wei, Jian-Ping Chen, Heng-Mu Zhang

**Affiliations:** 1 College of Agriculture and Biotechnology, Zhejiang University, Hangzhou, 310058 China; 2 State Key Laboratory Breeding Base for Zhejiang Sustainable Pest and Disease Control, Ministry of Agriculture Key Laboratory of Biotechnology in Plant Protection, Zhejiang Provincial Key Laboratory of Plant Virology, Institute of Virology and Biotechnology, Zhejiang Academy of Agricultural Sciences, Hangzhou, 310021 China; 3 Public Lab, Zhejiang Academy of Agricultural Sciences, Hangzhou, 310021 China; 4 Fujian Province Key Laboratory of Plant Virology, Institute of Plant Virology, Fujian Agriculture and Forestry University, Fuzhou, 350002 China

## Abstract

Virion distribution and ultrastructural changes induced by the infection of maize or rice with four different reoviruses were examined. *Rice black streaked dwarf virus* (RBSDV, genus *Fijivirus*), *Rice ragged stunt virus* (RRSV, genus *Oryzavirus*), and *Rice gall dwarf virus* (RGDV, genus *Phytoreovirus*) were all phloem-limited and caused cellular hyperplasia in the phloem resulting in tumors or vein swelling and modifying the cellular arrangement of sieve elements (SEs). In contrast, virions of *Rice dwarf virus* (RDV, genus *Phytoreovirus*) were observed in both phloem and mesophyll and the virus did not cause hyperplasia of SEs. The three phloem-limited reoviruses (but not RDV) all induced more flexible gateways at the SE-SE interfaces, especially the non-sieve plate interfaces. These flexible gateways were also observed for the first time at the cellular interfaces between SE and phloem parenchyma (PP). In plants infected with any of the reoviruses, virus-like particles could be seen within the flexible gateways, suggesting that these gateways may serve as channels for the movement of plant reoviruses with their large virions between SEs or between SEs and PP. SE hyperplasia and the increase in flexible gateways may be a universal strategy for the movement of phloem-limited reoviruses.

## Introduction

In plants, symplastic infection allows viruses to invade multiple types of cell, including mesophyll cells (MC), phloem parenchyma (PP), companion cells (CC) and sieve elements (SE)^1^. The different transport requirements of different cell types are catered for by a diversity of intercellular channels with specific functions at the cellular interfaces. These include the plasmodesmata (PD) on the MC-MC interface, the plasmodesmata pore unit (PPU) at the SE-CC interface and the sieve pore on the SE-SE sieve plate (SP) interface. These gateways provide multiple channels for the intercellular or systemic movement of plant viruses within their hosts. At the MC-MC interface, the PD are highly-specialized structures and transportation of non-cell autonomous proteins through them is active and selective^2^. In morphology, the PDs are intracellular openings between cells bounded by the plasma membrane (PM) with a modified ER membrane (referred to as the desmotubule, or DT) at the center of the structure. There is a small cytoplasmic sleeve or cavity between the PM and the DT and an intra-desmotubule space^3^. The selection and structural modification of the materials being transported is controlled by specific proteins on the DT and the membrane^4^. Callose (β-1,3-glucan) turnover on the CW around the entrance of PD can also regulate the permeability of the channel^5^. The capacity of PD to transport molecules is measured by their size exclusion limit (SEL). Morphological modifications of PD under different physiological states can be detected by transmission electron microscopy (TEM)^6^ or super-resolution fluorescent microscopy^7^. During leaf development, tissues undergo a transition from sink to source while the morphology of PD changes from simple to a branched complex to achieve a larger SEL and a more complex function^8^. At the opposing cellular interface of a heterograft union, PD are not usually completely rebuilt leading to a half-PD morphology with the CW not continuously punctured by PD channels^9^. When PD are hijacked by virus infection for facilitating intercellular movement, their SELs are enlarged to accommodate the macromolecular intruder^10^. When movement through PD occurs as a viral ribonucleoprotein complex (e.g. in tobamoviruses), the SEL increase is moderate and there is no observable morphological change in the PD^11^. In some other viruses, a viral-encoded movement protein (MP) forms a tubule within PD to guide intact virions passing through the PD and the SEL increase is then so great that a morphological change can be observed by TEM^11^. For example in comoviruses the DT structure is removed to accommodate a tubule over 40 nm wide^12,13^. To prevent or delay the pathogen passing through PD, plants employ enhanced callose deposition in PD to seal off the channel^10^.

Transportation through the PPU on the SE-CC interface involves a more intensive exchange of messages and materials than at the MC-MC interface and has to be highly selective^14,15^. The PPU is considered as a special type of PD with larger trafficking space and a larger SEL^16^, modified to be branched on the CC side but remaining single-stranded on the SE side^17^. Viral movement can also hijack the PPU: the intact virion of carrot red leaf virus (CtRLV) (26 nm in diameter) was observed in PPUs without leading to any structural change of the gateway that could be detected by TEM^18^.

Transport of macromolecules in sieve tubes is passive and non-selective^19^ and the sieve pore at the sieve plate (SP) between adjacent SEs has large orifices to maintain adequate flow^20^. Except for on SP, sieve pores were also located on the non-SP SE-SE interface, so called lateral sieve pore^7,21^. Both sieve plate pore (SPP) and lateral sieve pore are also developed from PD by remodeling to remove the DT impediment and generate a large opening^10,22^. CW modification of the polysaccharide component, such as sequential deposition and degradation of callose plays an important role in the permeability of the sieve pores^22^. Although DT is removed, ER is still retained in the sieve pore for facilitating symplastic transportation in phloem^7^. We recently reported a uniquely-structured channel, termed the flexible gateway^23^, on the SE-SE interface within tumors induced by *Southern rice black-streaked dwarf virus* (SRBSDV)^23^, a novel member of genus *Fijivirus*, family *Reoviridae*. This intercellular channel resembles PD in morphology but has a similar capacity to the sieve pore^23^. TEM shows a central pith structure and osmiophobic CW modification at the periphery resulting in very little open space within the channel but it has the capacity to accommodate the large virions of SRBSDV, about 80 nm in diameter^23^. These flexible gateways are not only concentrated on the sieve plate but are also frequent on the non-SP SE-SE interface of the SE hypertrophied region in SRBSDV-infected phloem but they are much less frequently observed in normal sieve tubes without hyperplasia.

We have now extended our study to compare the earlier results from SRBSDV with those from plant reoviruses representing all three plant-infecting genera in the family *Reoviridae*. Most members of the genera *Fijivirus*, *Oryzavius* and *Phytoreovirus* naturally infect monocotyledonous plants and induce galls, tumors, or vein swellings^24^ and all have icosahedral virions of 60–80 nm in diameter with no membrane envelope. The viruses studied were: (1), *Rice black-streaked dwarf virus* (RBSDV, genus *Fijivirus*) which causes dwarfing of both maize and rice, with white waxy tumor protrusions^25^ from the veins on the underside of maize leaves and swellings on rice stems; (2) *Rice gall dwarf virus* (RGDV, genus *Phytoreovirus*) which causes dwarfing and galls on the veins on the underside of rice leaves; (3) *Rice ragged stunt virus* (RRSV, genus *Oryzavirus*) which causes stunting, twisted leaves and splitting of the leaf margin and swelling of the veins on the underside of rice leaves; and (4) *Rice dwarf virus* (RDV, genus *Phytoreovirus*) which causes dwarfing and mild chlorosis (small flecks) on rice leaves and is the only known plant reovirus that does not induce tumors. Our findings provide more insight into the cellular distribution and movement patterns of plant reoviruses.

## Results

### RBSDV, RGDV and RRSV, but not RDV, are phloem-limited

**Figure 1:**
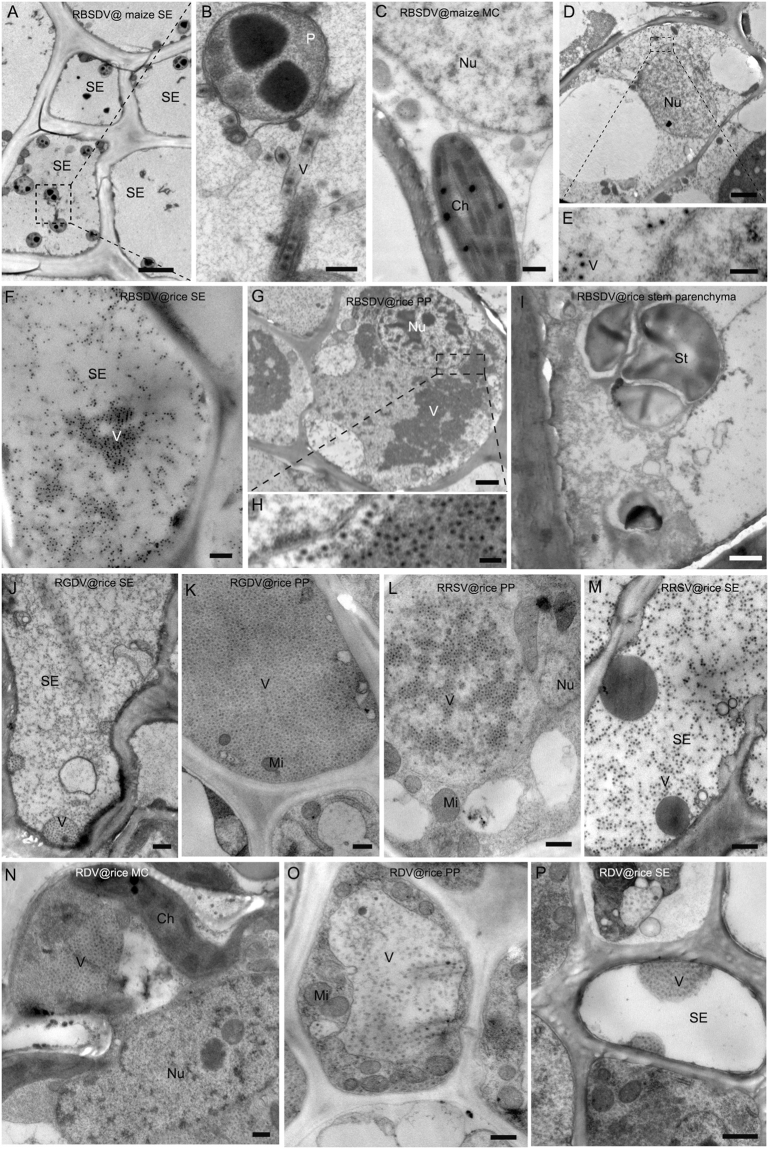
Cellular distribution of different reoviruses in plant hosts. (**A**–**E**) RBSDV in maize leaf. (**A**,**B**) RBSDV virus-like particles (VLP) in SE in infected phloem. (Bar in G = 2 μm; Bar in H = 200 nm). (**C**) No virion was detected in MCs from infected leaves. (Bar = 500 nm). (**D**,**E**) Virion distribution in PP in the infected phloem. (Bar in D = 2 μm; Bar in E = 200 nm). (**F**–**I**) RBSDV in rice stem. (**F**) Virion distribution in SE in the infected phloem. (Bar = 500 nm). (**G**,**H**) Virion distribution in PP in the infected phloem. (Bar in G = 2 μm; Bar in H = 200 nm). (**I**) No virion was detected in the stem parenchyma of infected plant. (Bar = 1 μm). (**J**,**K**) RGDV in rice leaf. (**J**) Virion distribution in SE in the infected phloem. (Bar = 500 nm). (**K**) Virion distribution in PP in the infected phloem. (Bar = 500 nm). (**L,M**) RRSV in rice leaf. (**L**) Virion distribution in PP in the infected phloem. (Bar = 500 nm). (**M**) Virion distribution in SE in the infected phloem. (Bar = 500 nm). (**N**–**P**) RDV in rice leaf. (**N**) Virion distribution in leaf MC infected with RDV. (Bar = 500 nm). (**O**) Virion distribution in PP in the infected phloem. (Bar = 500 nm). (**P**) Virion distribution in SE in the infected phloem. (Bar = 500 nm).

RDV virions were readily seen in the negatively-stained crude sap of both phloem and mesophyll tissues. By contrast, virions of RBSDV, RGDV and RRSV were abundant in sap from vascular tissues but rare or absent in mesophyll tissue extracts. These preliminary indications were confirmed by TEM observations. Double-layered virus-like particles (VLPs) were observed in the SE (Fig. 1A,B) and PP (Fig. 1D,E) of RBSDV tumors on maize but not in the xylem, MC (Fig. 1C) or in non-tumor phloem cells. Similar results were obtained from RBSDV-infected rice with VLPs present in tumor SE (Fig. 1F) and PP (Fig. 1G,H) but not in xylem or stem parenchyma (Fig. 1I) and rarely in the non-tumor phloem cells. RGDV and RRSV VLPs also appeared to be restricted to tumor phloem cells (Fig. 1J–M). By contrast, RDV VLPs were distributed in mesophyll (Fig. 1N), SE (Fig. 1O) and PP (Fig. 1P) cells, where they were located in the cytoplasm rather than in the nucleus.

### The phloem-limited reoviruses induced cellular hyperplasia and modified the pattern of cell arrangements in the phloem

**Figure 2:**
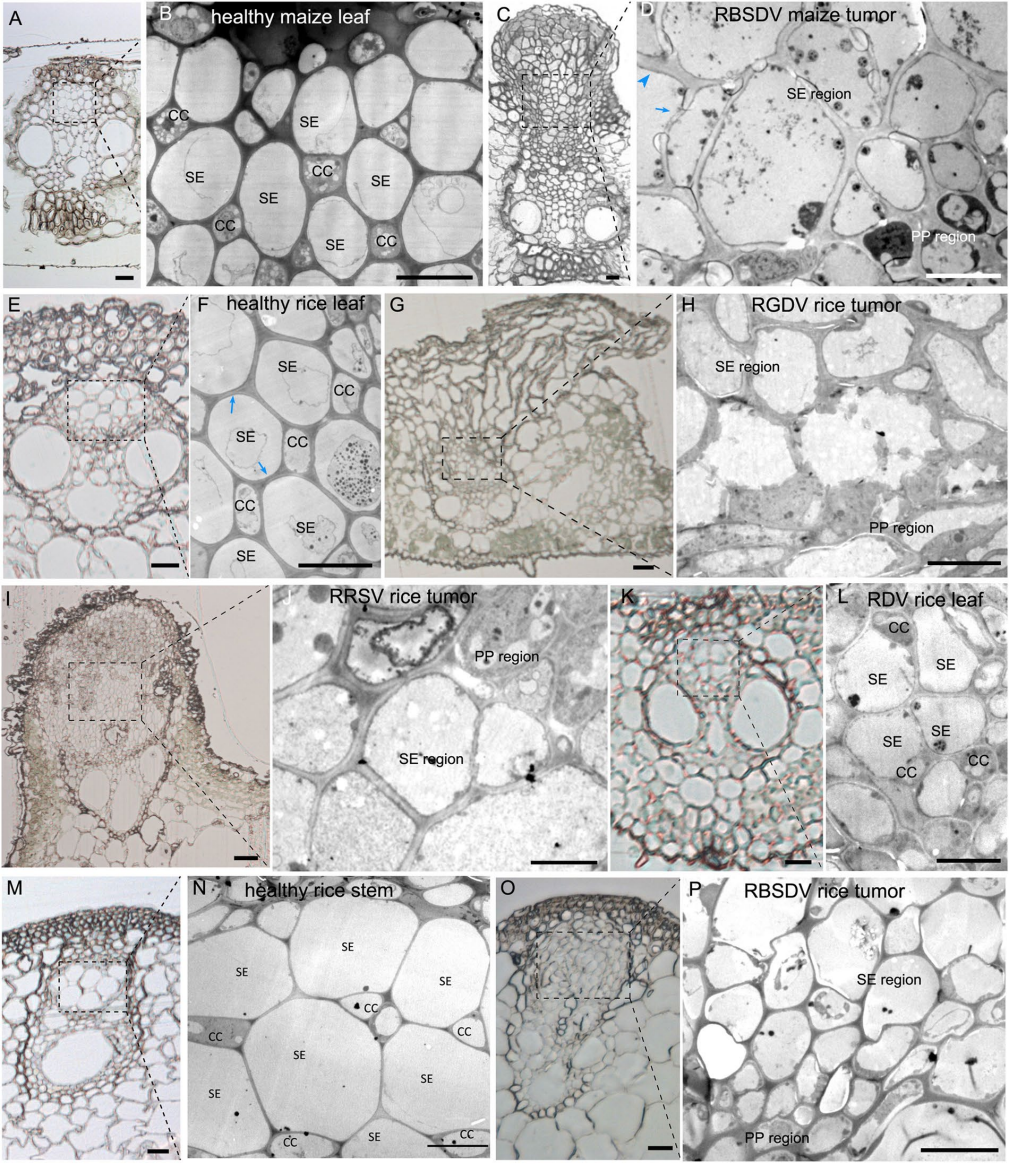
Re-organization of cells in plant-reoviral infected phloem. (**A**) Histological section of healthy maize leaf vascular bundle. (Bar = 20 μm). (**B**) Magnification of healthy maize leaf phloem in (**A**) under transmission electron microscopy (TEM) showing its cellular arrangement with a staggered SE-CC pattern. (Bar = 10 μm). (**C**) Histological section showing the cellular hyperplasia of the phloem in the infected vascular bundle causing the tissue to erupt through the epidermis as a tumor. (Bar = 20 μm). (**D**) Magnification of RBSDV-infected maize phloem in (**C**) under TEM showing how SEs are aggregated into exclusive regions without CC. (Bar = 10 μm). Most SEs in the tumor have thick CWs but thickening was not homogeneous (thickened part marked by blue arrowhead and the thin part by blue arrow). (**E**) Histological section showing the healthy vascular bundle of rice leaf. (Bar = 10 μm). (**F**) Magnification of healthy phloem of rice leaf in (**E**) under TEM showing its cellular arrangement with staggered SE-CC pattern. (Bar = 5 μm). (**G**) Histological section showing the cellular hyperplasia of the phloem in the infected vascular bundle which erupted through the epidermis as a tumor. (Bar = 20 μm). (**H**) Magnification of RGDV-infected rice phloem in (**G**) under TEM showing how SEs are aggregated into exclusive regions without CC. (Bar = 10 μm). (**I**) Histological section showing the cellular hyperplasia of the phloem in the infected vascular bundle which erupted from the epidermis as a swollen vein. (Bar = 20 μm). (**J**) Magnification of RRSV-infected rice phloem in (**I**) under TEM showing how SEs are aggregated into exclusive regions without CC. (Bar = 5 μm). (**K**) Histological section showing the non-hyperplasia of RDV-infected rice phloem. (Bar = 10 μm). (**L**) Magnification of RDV-infected rice phloem in (**K**) under TEM showing its cellular arrangement with staggered SE-CC. (Bar = 5 μm). (**M**) Histological section of healthy vascular bundle without hyperplasia. (Bar = 20 μm). (**N**) Magnification of healthy phloem in (**M**) under TEM showing its cellular arrangement with a staggered SE-CC pattern. (Bar = 10 μm). (**O**) Histological section showing the cellular hyperplasia of the phloem in the infected vascular bundle but without eruption into a tumor. (Bar = 20 μm). (**P**) Magnification of RBSDV-infected rice phloem in (**O**) under TEM showing how SEs are aggregated into exclusive regions without CC. (Bar = 10 μm).

We next examined the effects of virus infection on phloem development. The vascular bundles of healthy maize leaves consist of a xylem with vessels and supporting cells, and a phloem with SE-CC complexes and PP (Fig. 2A). The SEs and CCs are arranged in a staggered pattern, so that each SE is adjacent to at least one CC (Fig. 2B). There is a similar arrangement in the veins of rice leaves (Fig. 2E,F) and rice stems (Fig. 2M,N). The SEs account for 16–20% of all phloem cells and because SEs are separated by CCs, SE-SE interfaces account for only 9–14% of the total interfaces (Table 1).

**Table 1:** Characteristics of the sieve element (SE) cells of different tissues after reovirus infection. Figures are based on examination by TEM of 100 cells of each type, from each of 5 replicate plant samples.

	SE numbers as % of all phloem cells	SE-SE interfaces as % of total	Thick-walled SEs (%)	Frequence of SEs with flexible gateways	Frequence of flexible gateways between thick-walled SEs/thin-walled SEs
Healthy maize leaf	19.7 ± 1.36	13.5 ± 0.56	18.38 ± 7.65	0.6 ± 0.10	/*
Healthy rice leaf	19.1 ± 0.95	14.0 ± 1.28	15.18 ± 3.93	0.8 ± 0.08	/*
Healthy rice stem	16.0 ± 1.79	9.4 ± 0.84	13.50 ± 6.40	0.6 ± 0.01	/*
RBSDV maize leaf	33.9 ± 2.47	26.1 ± 1.14	96.73 ± 2.77	67.7 ± 1.04	82.5 ± 23.54
RBSDV rice stem	33.0 ± 1.73	24.9 ± 1.04	79.62 ± 6.71	40.8 ± 1.56	24.8 ± 11.95
RGDV rice leaf	32.9 ± 1.73	28.3 ± 1.38	83.98 ± 2.96	41.0 ± 0.57	25.3 ± 15.53
RDV rice leaf	19.2 ± 1.31	11.4 ± 1.18	15.08 ± 1.23	0.6 ± 0.03	/*
RRSV rice leaf	36.2 ± 1.01	27.8 ± 1.98	83.57 ± 3.85	75.1 ± 1.83	27.0 ± 8.66

*Not determined because there were few flexible gateways.

The histological and cellular arrangements in plants infected by the phloem-limited reoviruses were quite different. RBSDV-induced tumors in maize leaves were composed of hypertrophied cells or hyperplasia of the phloem (Fig. 2C), which ruptured the epidermis, while the size and arrangement of the xylem was unaffected. The normal staggered pattern of SE-CC was absent and proliferated SEs aggregated into exclusive regions without CCs, while the PP cells were also aggregated (Fig. 2D). RBSDV-infected rice stems had swellings rather than waxy tumors, and the cellular hyperplasia in the phloem did not burst the epidermis but appeared to occur internally, compressing the xylem (Fig. 2O) compared to the healthy control (Fig. 2P). In addition, the cells of RBSDV-infected phloem were smaller than in healthy stems (Fig. 2O,P). The galls of RGDV-infected rice leaves were also composed of hyperplastic phloem (Fig. 2H) erupting through the epidermis (Fig. 2G) and similar effects were seen in the swollen veins of RRSV-infected rice leaves (Fig. 2I,J). In all these cases, SEs accounted for a significantly larger proportion of the phloem cells and, because SEs were aggregated, the proportion of SE-SE interfaces rose to approximately twice that in healthy plants (Table 1).

In contrast, there was no cellular hyperplasia in the vascular bundle of RDV-infected leaves (Fig. 2K) where the staggered SE-CC pattern (Fig. 2L) was similar to that of the healthy control (Fig. 2E,F), the proportion of SEs was similar to that of healthy tissue and the proportion of SE-SE interfaces was slightly less than in the control (Table 1).

### All three phloem-limited reoviruses increased the proportion of thick-walled SEs and the frequency of flexible gateways

We next examined the cell walls and intercellular connections within the modified phloem of infected plants. Phloem SEs of maize are of two types, differing significantly in the thickness of their cell walls^26^. In our samples, only 13–19% of the SEs of healthy plants were thick-walled (defined as CW thickness across the SE and adjacent cell >500 nm), whereas in the infected plants the great majority had thick walls (Table 1). However, single SEs in tumors often had a mixture of both thick (Fig. 2D, blue arrowhead) and thin walls (Fig. 2D, blue arrow) whereas cells in normal phloem had a homogeneous thickening pattern within a single cell (Fig. 2F, blue arrow).

**Figure 3:**
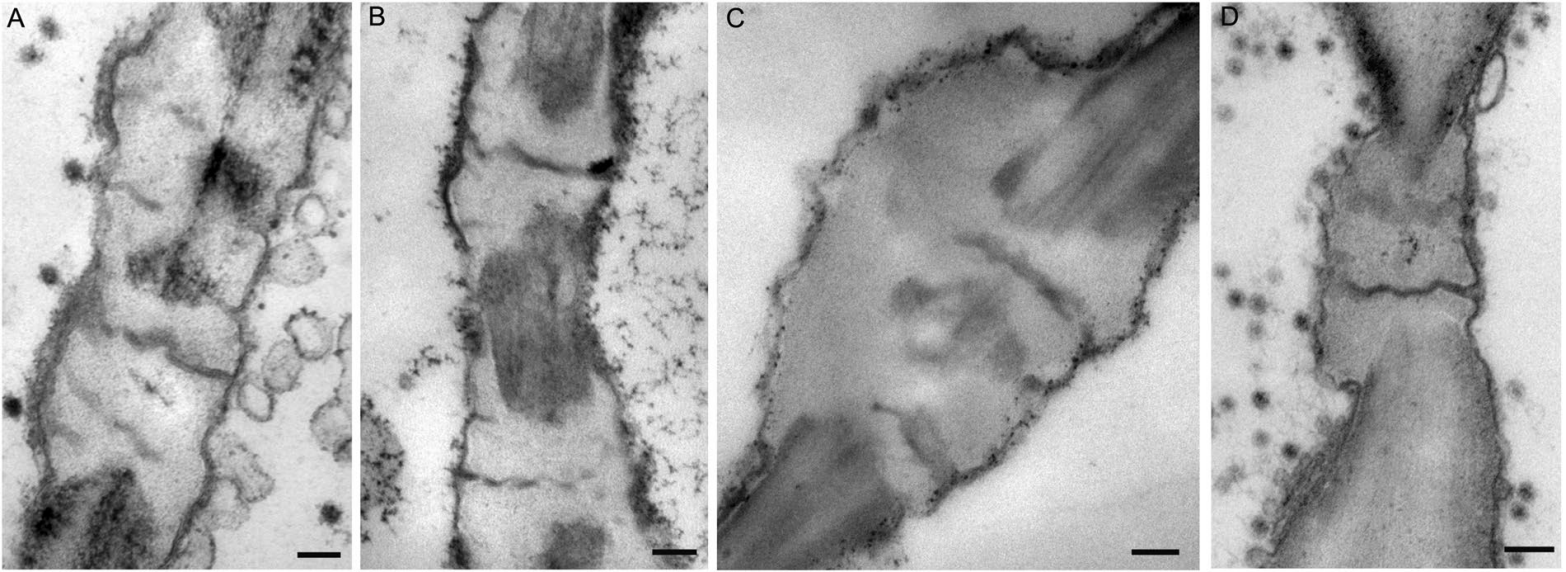
Morphology of flexible gateways in rice. (**A**) RBSDV-infected phloem of rice stem. (Bar = 200 nm). (**B**) RGDV-infected phloem of rice leaf. (Bar = 200 nm). (**C**) RDV-infected phloem of rice leaf. (Bar = 200 nm). (**D**) RRSV-infected phloem of rice leaf. (Bar = 200 nm).

**Figure 4:**
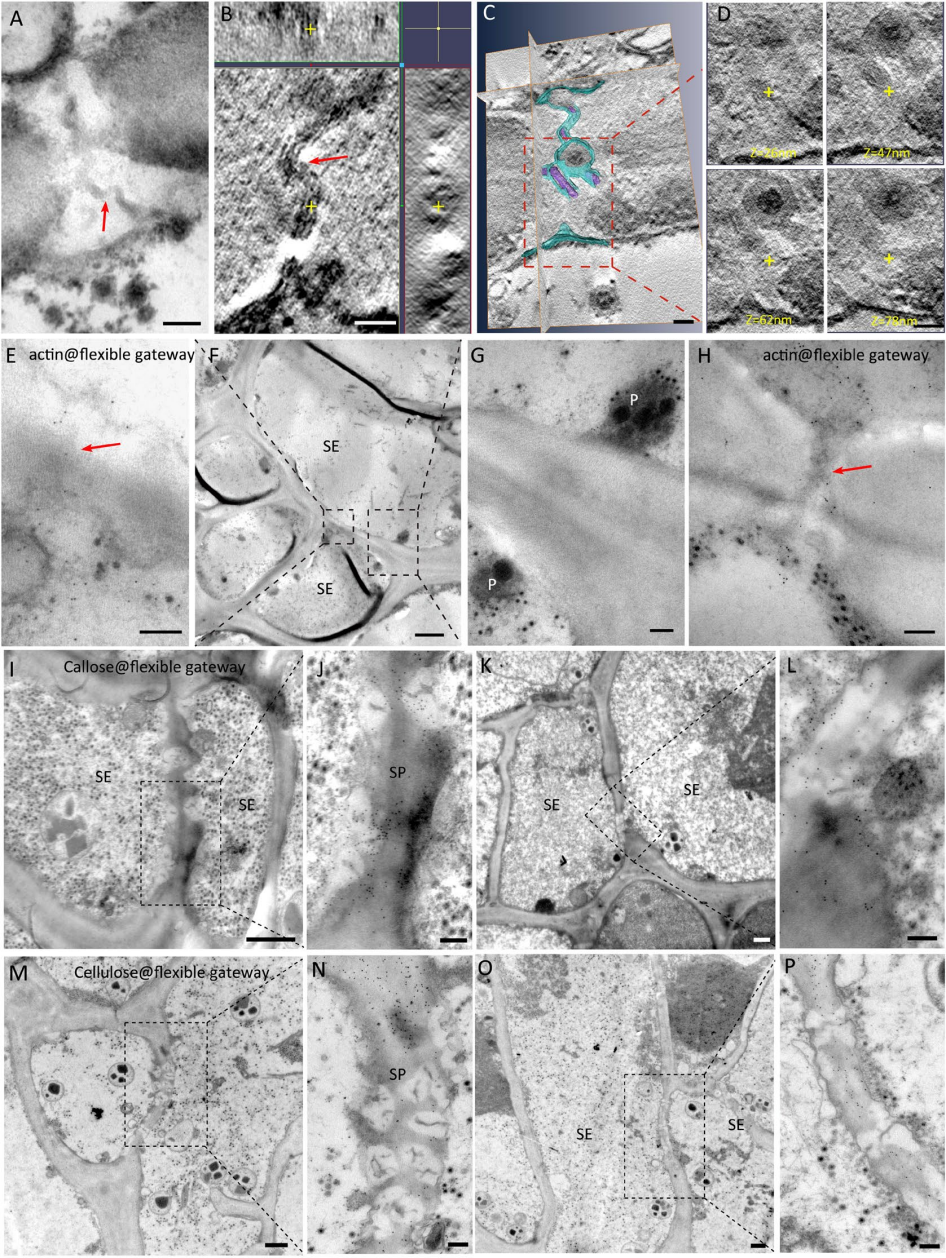
Properties of the flexible gateway in maize tumor revealed by TEM. (**A**) Flexible gateway observed on the SE-SE interface of tumor in RBSDV-infected maize, showing the desmotubule (DT)-like structure marked with red arrow. (Bar = 100 nm). (**B**) Electron tomography showing the existence of a DT-like structure (yellow cross in three different dimensions) and membrane in the flexible gateway. (Bar = 50 nm). (**C**) Structural segmentation of the DT-like structure (rendered in purple) and the membrane (rendered in blue). (Bar = 50 nm). (**D**) Serial digital slices of the central cavity module at different z positions, (the same x-y coordinates for each z slice are marked with a yellow cross). (Bar = 50 nm). (**E**–**G**) Immuno-gold labeling of actin on flexible gateway (red arrow) at the SE-SE interface, (note SE specific plastid (**G**)). (Bar in F = 2μm, Bar in E, G = 200 nm). (**H**) Another example of actin labeling on flexible gateway (red arrow) (Bar = 200 nm). (**I**–**L**) Immuno-gold labeling showing that callose was deposited on the peripheral CW of flexible gateways, which were located on both the sieve plate (SP) interface (**I**) and the non-SP SE-SE interface (**K**). (Bars in I and K = 2 μm; Bars in J and L = 200 nm). (**M**–**P**) Probe-gold labeling showing that cellulose was absent from the peripheral CW of flexible gateways located on both the sieve plate (SP) interface (**M**) and non-SP SE-SE interface (**O**). (Bars in M and O = 2 μm; Bars in N and P = 200 nm).

SEs mostly connected with one another by the high capacity sieve pore or the flexible gateway. Flexible gateways occurred on the phloem SE-SE interfaces (both SP and non-SP) of all samples, including the infected ones, healthy maize and healthy rice. Their appearance was similar in all samples regardless of host or the presence of virus (Figs 3A–D and 4A) but their frequency was much greater in all samples where virus infection had caused phloem hyperplasia and was also much greater between adjacent thick-walled SEs than between thin-walled ones (Table 1).

### Virus-infected maize and rice have similar flexible gateways within their tumors

Detailed structural and cytochemical experiments were used to compare the properties of the maize flexible gateways with those previously described in rice. The maize flexible gateways were located on both the SP interface (Fig. 4M,N) and the non-SP SE-SE interface (Fig. 4O,P). Tomography analysis revealed the 3D structure of the central pith which could be observed in three different dimensions (the yellow cross in Fig. 4B). This central pith had a diameter of 5–8 nm, slightly larger than the membrane, and resembled compressed DT of PD. Immuno-gold labeling of actin on the central pith suggested that cytoskeleton protein was present in the channel (Fig. 4E–H) and a quantitative study confirmed that the density of labeling on the flexible gateway was similar to that on the cytoplasm (positive control) rather than to that on CW material (negative control) (Table 2). A membrane-like structure surrounding the central pith was attached to the inner wall, with no cytoplasmic sleeve to the central pith. As in rice tumors, the central pith (rendered with purple) and the membrane (rendered with blue) in some individual maize flexible gateways did not completely cross the CW (Fig. 4C,D). When a virion was present in the intercellular channel, it partially displaced the central pith module while the outer membrane extended around the virion (Fig. 4C). Gold labeling experiments using an antibody against callose and a probe against cellulose showed that callose was being deposited (Fig. 4I,L) and cellulose was being removed (Fig. 4M–P) from the CW surrounding both SP and non-SP flexible gateways. A quantitative study confirmed the significance of these effects (Table 2). These features of the flexible gateway in RBSDV-infected maize tumors were very similar to those reported for SRBSDV-infected rice^23^.

**Table 2:** Density of gold labeling for four different target molecules in flexible gateways and controls. Figures are means of 30 measurements except where shown. In all cases there was a significant difference in density between the flexible gateway and normal cell wall measurements (P < 0.01).

Target	Flexible gateway^a^	Cell wall	Cytoplasm	Vacuole
actin	93.5 ± 31.84	2.6 ± 1.17	102.9 ± 31.48	5.2 ± 3.50
callose	161.9 ± 57.85	5.0 ± 3.13	4.9 ± 3.42	1.0 ± 0.43
cellulose	24.5 ± 7.16	201.9 ± 41.54	4.2 ± 2.12	2.1 ± 1.11
	Flexible gateway	Cell wall	Virion scattered^b^	Virion crystal^c^
RBSDV-P10	64.68 ± 22.25	1.2 ± 0.42	19.4 ± 7.91	81.4 ± 13.40

^a^The areas used were the central pith of the flexible gateway for actin labeling; the electron translucent CW adjacent to the flexible gateway for both callose and cellulose labeling; virions involved in the flexible gateway for RBSDV P10 labeling (5 replicate measurements only). ^b^The area used included both scattered virions and cytoplasm. ^c^The area used included only the virion crystal.

**Figure 5:**
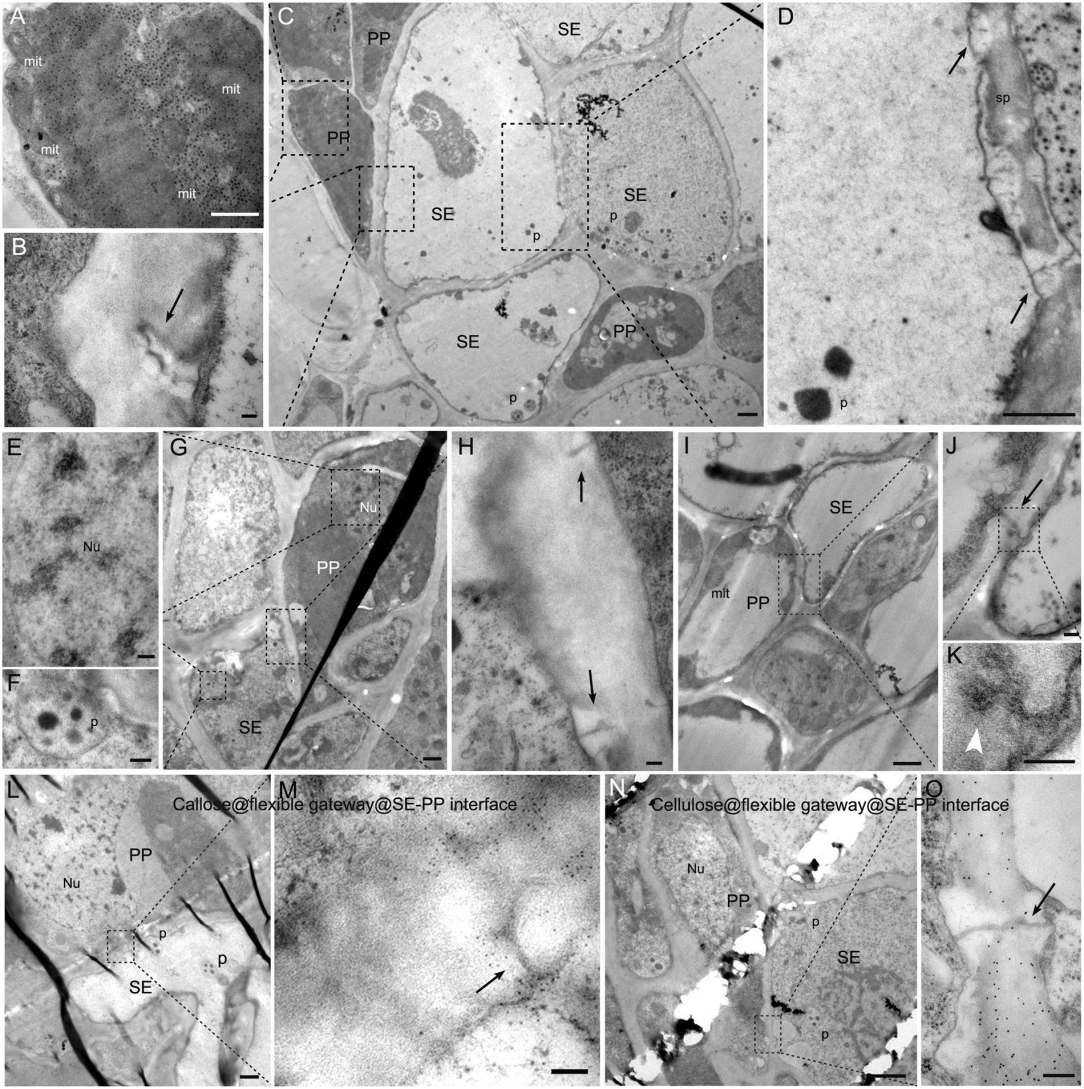
Flexible gateways on the SE-PP cellular interface. (**A**–**D**) Sequential magnification showing a flexible gateway on a SE-PP interface where the SE lacks cytoplasm (Bar in C = 2 μm). (**A**) PP verified by its cytoplasm and mitochondria (Bar = 1 μm). (**B**) Flexible gateway (black arrow) in the SE-PP interface with osmiophobic cell wall material (Bar = 100 nm). (**D**) SE verified by its SE-specific plastid and sieve plate where flexible gateways (black arrow) were also observed. (Bar = 1 μm). (**E**–**H**) Sequential magnification showing flexible gateway in SE-PP interface where the SE has retained its cytoplasm (Bar in G = 2 μm). (**E**) PP verified by its nucleus (Bar = 200 nm). (**F**) SE verified by its SE-specific plastid (Bar = 200 nm). (**H**) Flexible gateway (arrow) in the SE-PP interface with osmiophobic cell wall material (Bar = 100 nm). (**I**–**K**) Sequential magnification show the involvement of RRSV virus-like particle (white arrowhead) in flexible gateway located on a SE-PP interface. (Bar in I = 1 μm; Bar in J = 200 nm; Bar in K = 100 nm). (**L**,**M**) Gold labeling (5 nm) against callose on the flexible gateway at the SE-PP interface. (Bar in L = 1 μm; Bar in M = 100 nm). (**N**,**O**) Gold labeling (10 nm) against cellulose on normal cell wall but not on the osmiophobic cell wall of the flexible gateway located on a SE-PP interface. (Bar in N = 2 μm; Bar in O = 100 nm). SE, sieve element; PP, phloem parenchyma; mit, mitochondria; p, SE-specific plastid; sp, sieve plate; Nu, nucleus.

In addition, flexible gateways were also found on the cellular interfaces between SE and PP (Fig. 5A–H) where gold labeling experiments using an antibody against callose and a probe against cellulose also showed that callose was being deposited (Fig. 5L,M) and cellulose was being removed (Fig. 5N,O). They were never found on the other types of cellular interfaces. The gateways have a central pith structure of high electron density, looking like a compressed DT-like structure, surrounded by a deposit of osmiophobic CW material leaving scarcely any open space within the channels.

### Presence of large-sized reoviral virions within flexible gateways

**Figure 6:**
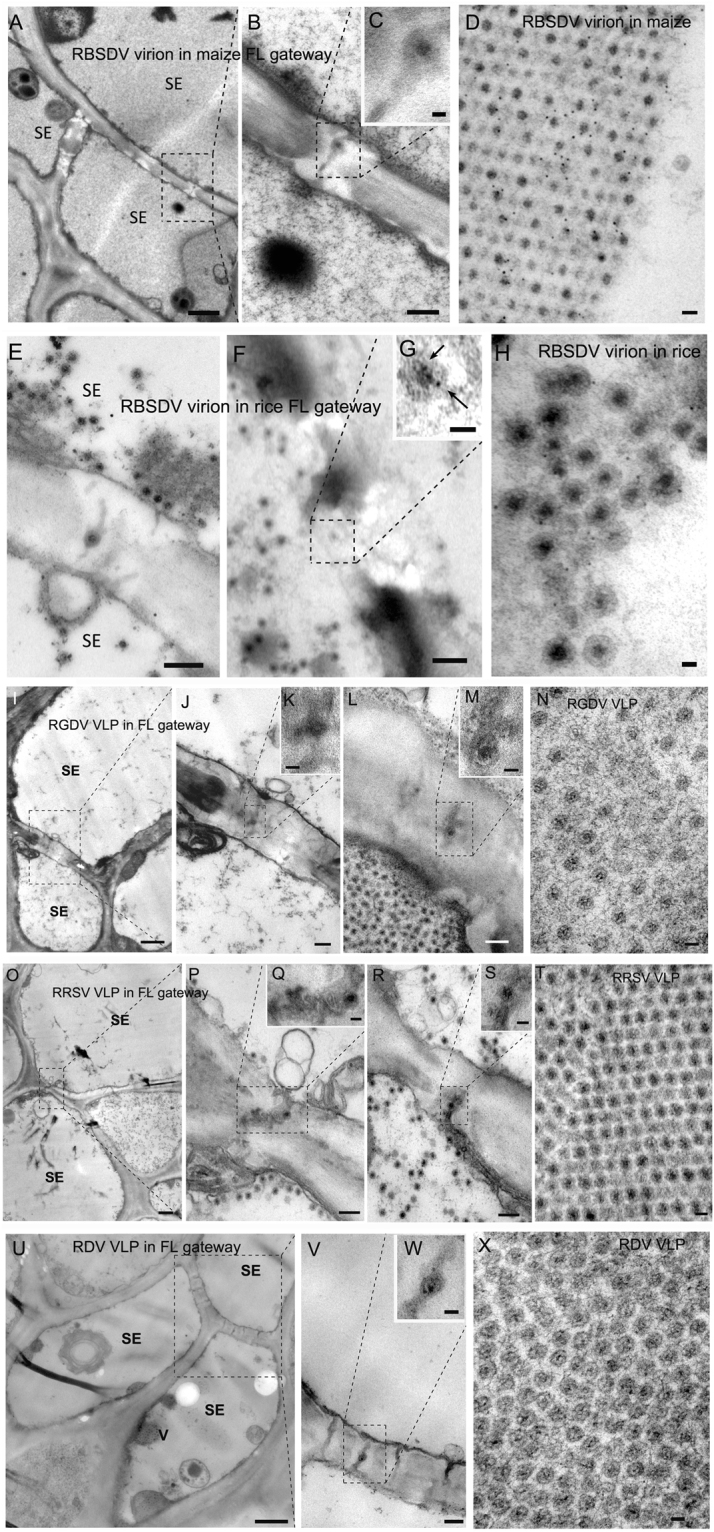
Virions in plants infected with reoviruses in the flexible gateway. (**A**–**C**) Sequential magnification of the flexible gateway on SE-SE interface of maize leaf and the presence of RBSDV VLP in the gateway. (Bar in A = 1 μm; Bar in B = 200 nm; Bar in C = 50 nm). (**D**) The morphology of RBSDV virion in crystals in an infected maize leaf. (Bar = 50 nm). (**E**) The flexible gateway located on SE-SE interface of rice stem and the presence of RBSDV VLP in the gateway. (Bar = 200 nm). (**F**,**G**) Sequential magnification of the VLP in the flexible gateway labelled with immuno-gold for the RBSDV outer capsid protein P10. Arrows show the gold particles labeled on virion. (Bar in F = 200 nm; Bar in G = 50 nm). (**H**) Immuno-gold labeling for RBSDV P10 confirming that the double-layered spherical particles are virions. (Bar = 50 nm). (**I**–**M**) Two examples of a flexible gateway located on the SE-SE interface of rice leaf, showing RGDV VLP within the gateway. (Bar in I = 1 μm; Bars in K and M = 50 nm; Bars in J and L = 200 nm). (**N**) The morphology of RGDV virions in crystals. (Bar = 50 nm). (**O**–**S**) Two examples of a flexible gateway located on SE-SE interface of rice leaf, showing RRSV VLP within the gateway. (Bar in O = 1 μm; Bars in Q and S = 50 nm; Bars in P and R = 200 nm). (**T**) The morphology of RRSV virion in crystals. (Bar = 50 nm). (**U**–**W**) Sequential magnification of the flexible gateway on SE-SE interface of rice leaf showing RDV VLP within the gateway. (Bar in U = 1 μm; Bar in V = 100 nm; Bar in W = 50 nm). (**X**) The morphology of RDV virions in crystals. (Bar = 50 nm).

**Figure 7:**
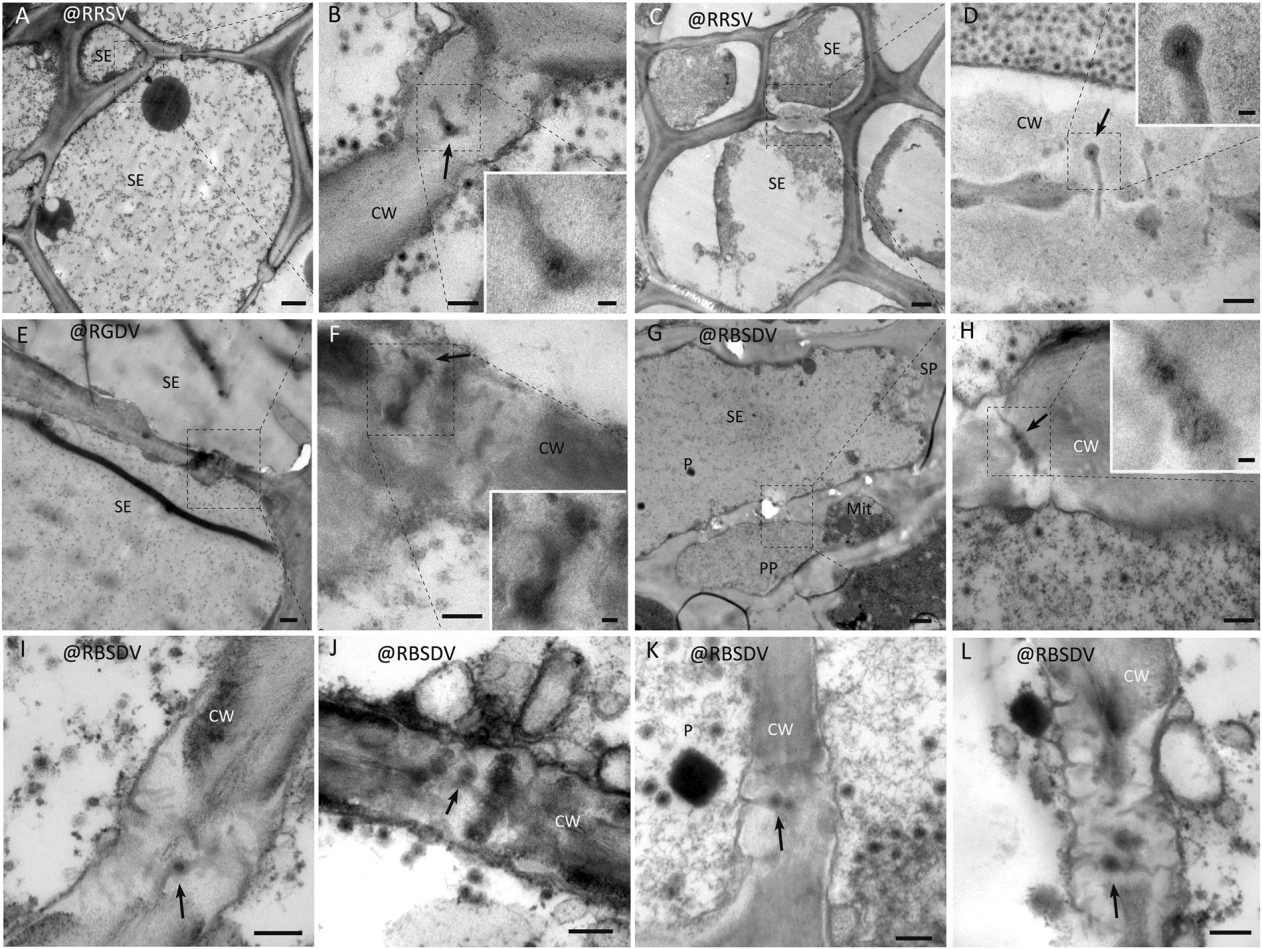
Universality of VLP involvement in flexible gateways of tumors induced by RBSDV, RGDV and RRSV. VLPs shown in (**G**,**H**) are located on the SE-PP interface. (Bars in **A**,**C**,**E**, and G = 1 μm; Bars in **B**,**D**,**F**, and **H**–**L** = 200 nm; Bars in small box = 50 nm).

In TEM, particles resembling VLPs were observed within the flexible gateways of all virus-infected samples. Examples are shown for RBSDV-infected maize leaf (Fig. 6A–C); RBSDV-infected rice stem (Fig. 6E), where the identity of the virion was confirmed by specific gold labeling against RBSDV P10 (Fig. 6F,G, arrow); RGDV-infected rice leaf (Fig. 6I–M); RRSV-infected rice (Fig. 6O–S); and RDV-infected rice (Fig. 6U–W). The presence of reoviral-like particles in flexible gateways was universal rather than occasional as revealed by repeated observation and it occurred at both the SE-SE and SE-PP interfaces (Fig. 7A–L).

A comparison of these structures with VLPs in other tissues also confirms their identity. RBSDV VLPs were spherical particles averaging 75 nm in diameter with double layers (Fig. 6D). They had a 50 nm osmiophilic core and a 20–30 nm shell and appeared similar in maize (Fig. 6D) and rice (Fig. 6H). Immuno-gold labeling using an antibody against the major capsid protein P10 showed that both the scattered (Fig. 6H) and aggregated crystal-like (Fig. 6D) VLPs were virions of RBSDV. The intensity of P10 labeling on virions in flexible gateways was similar to that on virion crystals (positive control) (Table 2). RGDV VLPs were also double-layered spheres, but a little smaller than those of RBSDV, averaging 65 nm in diameter (Fig. 6N), and with a smaller core with less osmiophilic material and a clear osmiophobic space between the two layers. RDV VLPs (Fig. 6X) resembled RGDV (Fig. 6N) rather than RBSDV (Fig. 6D and H). RRSV VLPs were also about 65 nm in diameter, but appeared to be similar to RBSDV in their morphology with no obvious space between the osmiophilic core and the outer shell (Fig. 6T).

## Discussion

Our results have shown that an abnormal phloem developmental pattern is characteristic of all the phloem-limited reoviruses, including RBSDV, SRBSDV, RGDV and RRSV. There is a significant increase in SE number but without typical CC. Proliferated SEs in tumors are aggregated as exclusive regions which results in more SE-SE interfaces and a greater frequency of flexible gateways. RDV is an exception among the reoviruses because it spreads to mesophyll cells and does not induce tumors or affect the number of flexible gateways. It seems that an association may exist between tumor induction and the increase in numbers of flexible gateways during reoviral infection. Tumor induction in the plant host is known to benefit infection by some viruses. For example, tumor induction contributes to the infection of geminiviruses by providing a particular microenvironment to meet the demands of vigorous viral DNA replication^27^. As a result, a series of host gene expression profiles are reprogrammed^28^. Infection of maize by the reovirus RBSDV also induces tumors in leaves and causes an alteration of some metabolic pathways^29^. Little is known about any advantage of tumor induction to reoviral infection but our observations suggest that, as with geminiviruses, tumor induction may assist viral replication by expanding the number of replication sites. To efficiently infect their plant hosts, viruses require a territory of well-connected cells for multiplication and movement^30^. Mesophyll tissue usually serves as such territory for the non-phloem limited viruses because the many mesophyll cells are connected to each other by abundant PD^31^. We speculate that normal phloem tissue may not be suitable to serve as the equivalent territory for phloem-limited viruses, because there are fewer cells supporting replication in the phloem. However, the SE hyperplasia and increased flexible gateways in the tumors induced by most plant reoviruses may provide the territory that is needed for efficient infection by these viruses. The phloem limitation of virus, which prevents viral infection in the mesophyll, might result from a barrier between SEs and adjacent phloem parenchyma (PP) cells^26^, either by a failure of the virus to suppress RNA silencing in the mesophyll or by a failure of MP function^31^.

Our results also show that flexible gateways occur in maize as well as in rice. Although they are present in healthy tissue, their frequency greatly increases following infection by the phloem-limited reoviruses. In morphology, the flexible gateway resembles the precursor of SPP^22^ as well as half PD^9^ but it is obviously different to mature SPP, typical PD and PPU, having a single compressed plasmalemma in the center and strong callose deposition nearby. In capacity, the flexible gateway resembles mature SPP but differs from PD and PPU in accommodating virions 80 nm in diameter. The flexible gateway is also unique in being present at both SE-SE and SE-PP interfaces. We have used the tentative name ‘flexible’ to describe the fact that the gateway is structurally occlusive but has the elastic capacity to accommodate large virions because of the localized deposition of a soft CW material, callose. Although the mechanism for developing flexible gateways is still uncertain, it appears that they can be stimulated by virus infection although only by those viruses that also induce hyperplasia. It is likely that, as with other intercellular channels, they develop from PD^16,22^. The presence of membrane-like structures which contain the cytoskeleton protein actin suggests that the ER and cytoskeleton may also participate in their biogenesis while CW remodeling involving callose turnover and cellulose evacuation is probably indispensable as in SPP development^32^.

It has been uncertain how plant reoviruses with their large virions move from cell to cell within plants. It has been reported that RDV moves through PD of MC interfaces as a viral ribonucleotide-protein (vRNP) complex and not as an intact virion because RDV P6 appears to function as a MP with ATPase and RNA-binding activities, with the ability to restore cell-to-cell movement of movement-defective PVX within leaves of *Nicotiana benthamiana* ^33,34^. However, the distribution of RDV in MC is unique and easily distinguished from the other rice reoviruses, all of which are phloem-limited as our studies confirmed.

The only clue to the movement of phloem-limited reoviruses has been our earlier report that the flexible gateway might provide a route for intact SRBSDV virions to move between SEs within virus-induced tumors^23^. We have now found that intact virions of RBSDV, RGDV and RRSV also occur within flexible gateways of maize or rice and it seems reasonable to suggest that this is how plant reoviruses generally move within the hypertrophied phloem. In addition, the present study also found the flexible gateways on SE-PP interfaces where they may provide channels for large capacity transportation. The presence of virions within a intercellular channel is usually regarded as evidence for viral movement, either intercellular^12^, or long-distance^1^. Based on our present observation, it seems reasonable to assume that the phloem-limited plant reoviruses, including RBSDV, SRBSDV, RGDV, RRSV, may employ a common pattern for their movement between SEs or between SE and PP via intact virions passing through the flexible gateways. Cellular hyperplasia of the phloem provides more flexible gateways connecting cells for their movement. In leaves, these viruses are absent because their virions are too large to move through PDs on the interfaces with MCs or other cell types. RDV appears to be an exception to this pattern as it forms vRNPs that can pass through PD and thus invade other cell types in the stem and leaves. It is interesting that we also observed VLPs of RDV in SEs and within the flexible gateways, suggesting that RDV might also move through the sieve tube as an intact virion, even if it moves as a RNP complex in other tissues.

## Methods

### Virus sources and antibodies

Reovirus-infected cereal plants were collected from sites in China and the viruses identified by RT-PCR and sequencing: RBSDV-infected maize from Zhejiang^25^, RBSDV-infected rice from Zhejiang^25^, RGDV-infected rice from Guangxi^35^, RRSV-infected rice from Fujian^36^ and RDV-infected rice from Fujian^37^. Cytochemical labeling studies used antibodies respective against plant actin (Agrisera, Sweden) and callose (Biosupplies, Australia). In addition, an enzyme-gold probe against cellulose (gifted by Prof. D.W. Hu) was used in the cytochemical labeling. An antibody against the P10 outer capsid protein of RBSDV was used to label the virion^24^. Secondary antibodies were 5 nm and 10 nm gold-IgG conjugates (Sigma, St Louis, MO).

### Microscopy and immuno-gold labeling

Morphological details of the phloem of virus-infected plants were photographed in a M165-FC stereomicroscope (Leica, Mannheim, Germany). At six weeks post infection, infected phloem samples were cut into small pieces, sequentially fixed by glutaraldehyde and osmium and embedded in Spurr resin (SPI supplies Inc. PA, USA) for cytopathological observation, or fixed by paraformaldehyde and embedded in Lowicryl K4M resin (EMS Inc. PA, USA) for immuno-labeling as previously described^23^. For histological studies, semi-thin sections of samples were cut by a UC6 microtome (Leica, Vienna, Austria) and observed under an Eclipse Ti optical microscope (Nikon, Tokyo, Japan).

To study virus distribution at the tissue level, leaf samples were separated into mesophyll and vascular tissue and crushed to produce crude sap. The sap was placed on formvar-covered copper grids, fixed with glutaraldehyde, negatively stained with 2% phosphotungstic acid (PTA, pH 6.7) and observed under an H-7650 electron microscope (Hitachi, Ibaraki, Japan) with a Gatan 830 CCD camera (Gatan, USA).

For cytopathological study, ultra-thin sections were sequentially stained with uranyl acetate and lead citrate and observed under the TEM. CW-associated polysaccharide was gold labeled on the Spurr resin sections as previously described and using a 200× dilution of callose antibody for 2 h and a 200× dilution of enzyme gold probe for 0.5 h^23^. Actin was gold labeled on the K4M resin sections using a 100× dilution of primary antibody for 2 h. Pre-immune serum was used as a negative control in the labeling experiments.

### Quantitative studies

The numbers of cells of different types that contained virions, the numbers of flexible gateways was assessed by image processing as described previously^23^ with some modification. After taking photographs at ×1000, the lengths of cells or CW was determined using ImageJ software^38^. To be recorded as a flexible gateway, the intercellular channel had to be located on the SE-neighbor cell interface, and to have a compressed DT-like structure in the center with large amounts of osmiophobic CW deposition around the gateway. The frequency of flexible gateways was calculated as the number of flexible gateways divided by CW length then multiplied by the number of SE-SE interfaces. This is a different formula to that used in the previous study^23^, to exclude effects of cell size. SE cells with CW thickness larger than 500 nm (across the SE and adjacent cell) were regarded as thick-walled. All quantitative studies were repeated at least five times in independent biological samples.

Quantitative studies of the intensity of gold labeling were done using automatic counts and area measurements made using the ‘IMRIS’ software package (Bitplane, Zurich, Switzerland). Subject areas (the central pith of flexible gateways for actin, the electron-translucent areas of CW adjacent to the flexible gateway for both callose and cellulose, VLPs in flexible gateways for RBSDV P10) were drawn manually and gold densities were determined from 30 repeats except for VLPs in flexible gateways (5 repeats because of their scarcity). A student’s *t*-test was used to determine the significance of differences in labeling density between flexible gateways and normal CW as control.

### Electron tomography and image processing

For ET reconstruction, sections (200 nm) were cut by a UC6 tomogram and placed onto 100-mesh carbon covered copper grids. Sample grids were first double stained by uranyl acetate and lead citrate and then treated with a suspension of 10 nm colloidal gold particles (Sigma, St. Louis, MO) for 5 min. The non-covalent binding of gold particles to sections provided fiducial markers for facilitating tomography alignments. Images were obtained automatically using a Tecnai F20 field emission gun (FEG) TEM (FEI, Amsterdam, Holland) at 200 kV accelerating voltage and recorded by a bottom-mounted FEI TVIPS F415MP CCD (size 4k × 4k) camera at the compression parameter of ‘binning = 2’. Tomographic data was collected automatically following the FEI software instructions from −60° to +60° at 2° step increments at a magnification of 15k. In tilting, images were consistently photographed with a defocus value of 6 μm under the same beam dose (calculation based on magnification, beam intensity and spot size). ET reconstruction was done by the IMOD ‘etomo’ program following the software instructions^39^. Iterative calculation of fine alignment was carried out to obtain a ‘residual error’ value less than 0.5. The final tomogram of the three-dimensional (3D) density map was generated by the simultaneous iterative reconstructive technique (SIRT) and was visualized by the ‘3dmod’ program of IMOD^39^. Image processing including segmentation and surface rendering was performed by the ‘AMIRA’ software (FEI, Hillsboro, USA), so that structures of interest were segmented from the density map and rendered in different pseudo-colors for analysis.
